# Screening for Problematic Internet Use May Help Identify Impulse Control Disorders in Parkinson's Disease

**DOI:** 10.1155/2019/4925015

**Published:** 2019-02-03

**Authors:** Márton Kovács, Attila Makkos, Dávid Pintér, Annamária Juhász, Gergely Darnai, Kázmér Karádi, József Janszky, Norbert Kovács

**Affiliations:** ^1^Doctoral School of Clinical Neuroscience, University of Pécs Medical School, Pécs, Hungary; ^2^Department of Neurology, University of Pécs Medical School, Pécs, Hungary; ^3^MTA-PTE Clinical Neuroscience MR Research Group, Pécs, Hungary; ^4^Institute of Psychology, University of Pécs, Pécs, Hungary; ^5^Institute of Behavioral Sciences, University of Pécs Medical School, Pécs, Hungary

## Abstract

**Background:**

Impulse control disorders in Parkinson's disease (PD) represent emerging problems with potentially devastating consequences. The standard screening methods for impulse control disorders are clinically imperfect. Although it is rarely reported, many patients utilize the Internet to fulfill their compulsive behaviors because of its easy accessibility. We designed a study to test the hypothesis that an active screening for excessive Internet use and Internet addiction might improve the sensitivity of identification of impulse control disorders.

**Methods:**

The standard screening method included the Questionnaire for Impulsive-Compulsive Disorders in Parkinson's Disease and the modified Minnesota Impulsive Disorders Interview. In the second round, the Problematic Internet Use Questionnaire was also assessed for detecting excessive Internet use.

**Results:**

While the standard approach identified 19 patients out of 106 (17.9%) with any type of impulse control disorders, screening for the problematic Internet use detected 29 patients with impulse control disorders (27.4%) having significantly better efficacy over the standard method (*p* = 0.004, the McNemar test).

**Conclusions:**

Our study suggests that the screening for problematic Internet use by the Problematic Internet Use Questionnaire is an effective, feasible, and easy-to-use add-on method for identifying PD patients with impulse control disorders more efficiently and probably at earlier stages.

## 1. Introduction

Although the impulse control disorders (ICDs) and related behaviors are increasingly recognized as the side effects of antiparkinsonian medications [1], recent studies demonstrated that compulsive gambling, buying, sexual and eating behaviors, and hobbyism may present in drug naïve Parkinson's disease (PD) patients [2], as well. The prevalence of ICDs varies in the range of 3.5%-42.8% being more frequent among males, younger patients, patients with younger disease onset and longer disease duration, and depressive symptoms [3]. Because PD patients with ICDs (PD-ICD) usually feel being ashamed of their compulsive behavior, the majority of them try to hide and deny their overwhelming and uncontrollable problems. Therefore, the correct and early detection of ICD symptoms is quite challenging. In many cases, the ICDs are recognized too late largely interfering with the everyday activities and resulting in devastating consequences in the personal, financial, occupational, and social status.

Therefore, there is a high clinical need for identifying PD-ICD at earlier stages. Numerous screening tools have been designed especially for recognizing PD-ICD (e.g., Questionnaire for Impulsive-Compulsive Disorders in Parkinson's Disease, QUIP [4], and modified Minnesota Impulsive Disorders Interview, mMIDI) [5]. Although these instruments have acceptable specificity and sensitivity at the group level, the identification of PD-ICD patients is far from the desired state at the individual level. To improve the sensitivity of screening methods, numerous centers use a combination of a self-assessment screening instrument (e.g., QUIP) and an interview-based instrument assessed by trained professionals (e.g., mMIDI) [6]. Despite these efforts, one can see a subtle number of PD-ICD patients who previously passed successfully the screen for ICDs having zero points on QUIP and mMIDI. Based on these false negative cases, one can state that the current approaches for screening for PD-ICD might be comprehensive yet imperfect. Therefore, new approaches should be incorporated into clinical practice to increase the accuracy of detection.

Analyzing the history and behavior of our PD-ICD patients, we recognized that the majority of them performed their compulsive gambling and sexual behavior on the Internet. Moreover, some patients with compulsive buying preferred online shopping over the traditional shopping methods. We hypothesize that many PD-ICD patients frequently use the Internet to satisfy their desire because the online gambling, porn, and shopping sites are easily accessible, and this way, they can hide their compulsive behavior and remain, at least partly, anonymous. Moreover, the Internet-based ICD activities do not require heavy physical demand, which is another advantage for those who have any physical handicaps.

Internet addiction appears to be a common disorder involving online and/or offline computer usage [7]. Internet addiction may be classified as a compulsive-impulsive spectrum disorder characterized by four components: excessive use, emotional withdrawal, tolerance (needs of more hours of use, etc.), and negative repercussions (arguments, lying, social isolation, etc.) [8]. Although ICD problems are adequately addressed in the recently published 5th edition of the Diagnostic and Statistical Manual DSM-V [9], Internet addiction is not included [10]. Internet addiction is often resistant to treatment entailing significant risks, having high relapse rates and making comorbid disorders less responsive to treatments [8].

We propose that the overuse of Internet or Internet addiction per se might be an indicator for the presence of ICDs, and consequently, the additional screening for the problematic Internet use might help identify PD-ICD patients at earlier stages and higher sensitivity. In our previous work on healthy volunteers, we demonstrated that people are more likely to confess their Internet addiction or Internet overuse than admit their other compulsive behaviors (e.g., gambling) [11]. Therefore, we assume that PD-ICD patients will less likely try to hide this behavior from the medical staff at screening. However, these assumptions need further confirmation.

Taking into consideration the presumable link between Internet overuse and ICDs in PD, we designed a prospective study to test the hypothesis that a screening for excessive Internet use or Internet addiction might improve the identification of PD-ICD patients.

## 2. Materials and Methods

In this study, 150 consecutive PD patients fulfilling the UK Brain Bank criteria without known ICD were enrolled. The patients were recruited and treated at the University of Pécs by neurologists specialized in movement disorders. Each subject gave written informed consent in accordance with the ethical approval of the Regional and Institutional Ethical Committee (3617.316-24987/KK41). Each patient underwent the screening for ICD in the following sequence:

- The comprehensive neuropsychological examination (including the validated Hungarian versions of the Montreal Cognitive Assessment, MoCA [12, 13]; Montgomery-Asberg Depression Rating Scale, MADRS [14]; Parkinson Anxiety Scale, PAS [15]; and Lille Apathy Rating Scale, LARS [16]) for measuring cognition, depression, anxiety, and apathy was assessed
- Patients were asked to rate the QUIP [4]. QUIP is a self-rating questionnaire with 15 items, which include 3 sections with 5 questions each: Section 1 is for assessing the four ICDs reported in PD (gambling, buying, sexual, and eating behaviors); Section 2 is for measuring other compulsive behaviors (punding, hobbyism, and walkabout); and Section 3 is for assessing compulsive medication use [4]
- Knowing the findings of the neuropsychological assessments and the QUIP, a highly experienced healthcare professional assessed the mMIDI [5]. The modified Minnesota Impulsive Disorders Interview [5] includes 5 modules measuring different ICDs: compulsive buying, compulsive gambling, compulsive sexual behavior, compulsive eating, and pounding behavior. Each module has a gateway question, and if the patient gives an affirmative answer, it was followed by additional questions about the actual ICD
- Based on the DSM-V diagnostic criteria for gambling disorder [9] and binge eating [9] and the proposed criteria for compulsive sexual behavior and compulsive buying [17] and dopamine dysregulation syndrome [18], the patients were categorized as either having or not having ICD problems. This categorization is referred to as the standard approach in the manuscript
- The International Parkinson's Disease and Movement Disorders Society sponsored version of Unified Parkinson's Disease Rating Scale, MDS-UPDRS [19], and the Unified Dyskinesia Rating Scale, UDysRS [20], if applicable, were assessed. The purpose of these tests was not only the phenomenological description of patients but also taking their mind off the topic of ICDs
- Subsequently, the patients were asked to rate their Internet use (including smartphones, tablets, laptops, and computers) by the self-rated Problematic Internet Use Questionnaire (PIUQ) [21]. Hitherto, there are no clear diagnostic criteria for the Internet addiction [9, 10]. Therefore, it is highly recommended to measure excessive Internet use with a continuous questionnaire [11]. We chose the Problematic Internet Use Questionnaire [21] because its structure tightly adheres to the proposed diagnostic criteria for Internet addiction [21, 22]. PIUQ was created based on the clinimetric and psychometric analysis of Young's Internet Addiction Test [23] independently validated by several groups [24–26]. The 3-factor construct of the PIUQ has also been generally supported [21, 24, 25] and consistently labeled “Obsession” (i.e., obsessive thinking about the Internet and mental withdrawal symptoms caused by the lack of Internet use), “Neglect” (i.e., neglect of basic needs and everyday activities), and “Control disorder” (i.e., difficulties in controlling Internet use). The PIUQ contains 18 self-rated items [21]. Another advantage of the PIUQ is its free-to-use license (available in http://demetrovics.hu/en/questionnaires.html). A total score exceeding 41 points suggests Internet addiction [25]
- Subsequently, the same health care professional reassessed the mMIDI knowing the results of the QUIP and PIUQ. If excessive Internet use was detected, specific questions on the Internet-related habits were also asked (e.g., hours spent with browsing and naming the frequently browsed websites). Internet addiction was diagnosed based on the proposed criteria [22], whereas Internet overuse was defined as the excessive use of the Internet not fulfilling the criteria for Internet addiction. Again, the patients were categorized as either ICD positive or negative (standard+PIUQ approach)

To compare the two ICD screening methods (standard vs. standard+PIUQ approaches), nonparametric tests were used (IBM SPSS, version 24.002, Armonk, NY). For correlation, Spearman's rank correlation coefficients were calculated. The values of correlation coefficients (*r*_*S*_) can indicate weak (0-0.299), moderate (0.300-0.599), and high (0.600-1.000) associations [27].

## 3. Results

Out of 150 PD patients screened, only 106 patients (70.7%) reported regular Internet use. Subsequently, the data of these Internet users were analyzed only. The baseline characteristics are reported in Table 1. The UDysRS was assessed only on those patients who experienced motor complications (*n* = 50, 47.2%).

Based on the standard evaluation approach, we identified three patients with pathological gambling. However, we found six additional patients with online gambling problems using the standard+PIUQ approach. Therefore, the standard+PIUQ approach performed statistically significantly better (*p* = 0.031, the McNemar test). The gambler PD-ICD patients identified by only the standard+PIUQ method had shorter ICD disease duration (6.2 ± 3.4 months vs. 18.4 ± 7.6 months, *p* < 0.05), milder symptoms (mMIDI score: 3.2 ± 1.4 vs. 6.5 ± 3.6 points, *p* < 0.05), and seemingly less serious consequences than the patients picked by the standard approach.

Compulsive sexual problems were recorded in two cases with the standard approach. Based on Internet overuse, another six patients were diagnosed with hypersexuality-type ICDs. Again the standard+PIUQ screening approach was superior (8 vs. 2 patients, *p* = 0.031, the McNemar test). Patients picked by the PIUQ had apparently milder problems and consequences associated with the ICDs.

As far as the compulsive eating was concerned, both screening methods identified the same number of patients.

The standard approach demonstrated compulsive buying in seven subjects, whereas the standard+PIUQ method picked eight patients (*p* = 1.000, the McNemar test).

Hobbyism, punding, and other compulsive problems were identified in seven instances by QUIP and mMIDI alone. Of note, none of the patients reported Internet addiction per se. Using the standard+PIUQ method, a total of 13 patients were picked. Out of these patients, 5 had Internet addiction syndrome per se. Therefore, the standard+PIUQ screening process is superior in identifying hobbyism spectrum ICDs (*p* = 0.031, the McNemar test).

No one had a dopamine dysregulation syndrome in our cohort of PD patients fulfilling the proposed criteria [18].

While the standard approach identified 19 patients out of 106 (17.9%) with any type of ICD, the standard+PIUQ screening process picked 29 patients (27.4%, Supplementary Materials). Therefore, the standard+PIUQ method had a significantly better efficacy (*p* = 0.004, the McNemar test).

In PD-ICD patients, not only the overall PIUQ score was higher (32.2 ± 5.4 vs. 19.2 ± 0.4, *p* < 0.001) but also the nonmotor experiences of daily living MDS-UPDRS and the dopamine agonist Levodopa equivalent dosage (Table 2).

Using Spearman's correlation, we did not find any correlation between apathy (LARS) and ICDs. However, both depression (MADRS) and anxiety (PAS) had a moderate correlation with the ICDs (rho = 0.344 and 0.370, respectively, both *p* < 0.05).

## 4. Discussion

Our pilot study suggests that the screening for problematic Internet use by PIUQ is an effective, feasible, and easy-to-use add-on method for identifying PD-ICD patients more efficiently and probably at earlier stages. Based on our results, compulsive gambling (especially online gambling and betting), hypersexuality, hobbyism/punding, and Internet addiction per se can be more efficiently identified. Although the statistical significance was not met, the screen for Internet overuse may also be helpful in diagnosing compulsive buying.

To check the novelty of this approach, we performed a PubMed search (keywords: “Parkinson's disease” and “Internet”) on March 15, 2018. Out of 162 matches, we identified only eight relevant publications. According to Weintraub and Claassen, Internet addiction can be categorized as a form of compulsive hobbyism [28]. Many teams [29, 30] share the view that Internet addiction is part of the hobbyism spectrum. Although numerous large-scale and multicenter studies aimed at evaluating PD-ICD, none of them assessed the problematic Internet use [1].

Pathological gambling in the “traditional” bingo halls may be also associated with Internet overuse [31]. On the other hand, numerous case series demonstrated that some patients tend to play online games, which is also linked to Internet overuse [32, 33]. While the compulsive “traditional” gambling in PD is well described, the use of the Internet to gamble is a less described emerging problem [33]. Its importance may also be highlighted by the fact that online gambling is not only described in PD-ICD but also in subjects with restless legs syndrome and prolactinoma treated by dopamine agonists [34].

The prevalence of Internet addiction in PD per se is less frequently described. Fan et al. revealed a single subject out of 312 Chinese PD patients (0.3%) with Internet addiction using mailed self-report screening questionnaires [30]. The single prospective study on the systematic evaluation of Internet addiction in PD included 29 PD-ICD patients, 20 PD patients without ICDs, and 19 healthy controls [35]. Shapira et al. utilized the Yale-Brown Obsessive Compulsive Scale adapted for Internet use (Y-BOCS-Internet) and the proposed criteria for Internet addiction [22] simultaneously. The PD-ICD patients had significantly higher scores on the Y-BOCS-Internet scale, spent more time on the Internet, and described significantly more effort to resist Internet use and its interference with their life functioning compared to PD patients without ICD [35]. Moreover, the majority of PD-ICD patients assessed a combination of online gambling, betting, shopping, auction, and pornography sites regularly and compulsively. Therefore, their results suggest that PD-ICD patients have a relatively increased tendency towards excessive Internet use compared to those without ICDs, which might support our theory that the active screening for Internet addiction and overuse may help better identify PD-ICD patients.

Although the screening for problematic Internet use is a novel and promising method for identifying PD-ICD, the authors are aware of some limitations. Because a considerable portion of patients (in our sample 44 out of 150, 29.3%) does not use the Internet, in their cases, the application of PIUQ does not have any additive value. Another limitation may be that the PIUQ has never been formally tested on PD population previously. Although our pilot study may be encouraging, the PIUQ seems to be helpful in identifying only certain ICD problems and not all of them. Moreover, a larger-scale (preferably multicenter) data is lacking on the regular use of PIUQ in PD.

## 5. Conclusions

Our pilot study supports the idea that the active screening for Internet overuse or addiction in a PD population may help identify ICD problems with higher accuracy and presumably at earlier stages than the standard approach. Further, preferably longitudinal and multicenter, studies are required to more precisely determine the accuracy of this screening approach.

## Figures and Tables

**Table 1:** The demographic and disease-specific characteristics of the study population reporting regular Internet usage (*n* = 106).

		Mean/count	Standard deviation/percentage	Median	Percentile 25	Percentile 75
Age (years)		71.0	0.0	70	65	75
Sex	Male	64	60.4%			
	Female	42	39.6%			
Education (years)		12.5	3.4	12	11	16
Disease duration (years)		10.0	3.6	10	3	13
Levodopa duration (years)		6.3	4.4	5	3	9
Disease subtype	Tremor-dominant	40	37.7%			
	Rigid-akinetic	40	37.7%			
	Mixed	26	24.6%			
Disease severity	Mild (HYS 1 & 2)	66	62.3%			
	Moderate (HYS 3)	22	20.7%			
	Severe (HYS 4 & 5)	18	17.0%			
Levodopa usage		60	56.6%			
Dopamine agonist usage		65	61.3%			
Monoamine oxidase inhibitor usage		18	16.9%			
Catechol-O-methyltransferase inhibitor usage		23	21.7%			
Anticholinergic usage		0	0%			
Deep brain stimulator usage		20	18.9%			
LED		384.2	509.8	300	0	600
Dopamine agonist LED		108.2	170.6	40	0	160
Total LED		628.7	1085.2	400	100	822
Lille Apathy Rating Scale		-24.0	6.8	-26	-28	-21
Montgomery-Asberg Depression Rating Scale		10.0	5.4	10	6	14
Montreal Cognitive Assessment		24.2	3.4	25	22	28
Parkinson Anxiety Scale		11.4	7.3	11	7	17
MDS-UPDRS nM-EDL		11.5	6.5	11	7	15
MDS-UPDRS M-EDL		12.1	8.0	12	5	18
MDS-UPDRS ME		28.8	12.8	28	19	38
MDS-UPDRS MC		2.9	2.7	2	0	4
MDS-UPDRS total score		55.1	23.7	53	36	73
UDysRS part 1		8.1	8.6	6	0	15
UDysRS part 2		3.8	3.1	4	1	6
UDysRS part 3		7.2	3.8	5	0	3
UDysRS part 4		1.4	2.0	0	0	3
UDysRS total score		20.5	13.3	16	5	22
PDQ-39 SI		20.5	14.4	18.0	10.0	30.0

HYS = Hoehn-Yahr stages; LED = Levodopa equivalent dosage; MDS-UPDRS = Movement Disorders Society sponsored version of Unified Parkinson's Disease Rating Scale; MDS-UPDRS MC = motor complications (part IV of MDS-UPDRS); MDS-UPDRS ME = motor examination (part III of MDS-UPDRS); MDS-UPDRS M-EDL = motor experiences of daily living (part II of MDS-UPDRS); MDS-UPDRS nM-EDL = nonmotor experiences of daily living (part I of MDS-UPDRS); PDQ-39 SI = 39-item Parkinson's Disease Questionnaire Summary Index; UDysRS = Unified Dyskinesia Rating Scale (measured only on patients having dyskinesia, *n* = 50).

**Table 2:** Comparison between Parkinson's disease patients with and without impulse control disorders (PD-ICD and No ICDs, respectively).

	No ICDs					PD-ICD					Significance
	Mean	Standard deviation	Median	Percentile 25	Percentile 75	Mean	Standard deviation	Median	Percentile 25	Percentile 75	
PIUQ Obsession subscale	6.2	0.2	6	6	6	10.2	2.4	9	6	10	*p* < 0.001
PIUQ Neglect subscale	6.3	0.2	6	6	6	11.5	1.8	11	8	11	*p* < 0.001
PIUQ Control subscale	6.3	0.2	6	6	6	10.5	2.2	10	6	10	*p* < 0.001
PIUQ total score	19.2	0.4	18	18	18	32.2	5.4	31	22	29	*p* < 0.001
mMIDI total score	0.8	5.4	0	0	1	4.4	7.1	0	0	6	*p* < 0.05
PDQ-39 SI	19.5	13.9	18	8	28	23.2	15.7	19	13	32	
MDS-UPDRS total score	55.3	23.5	53	36	74	54.8	24.8	53	36	66	
MDS-UPDRS nM-EDL	10.7	5.9	10	6	15	13.6	7.6	14	7	19	*p* < 0.05
MDS-UPDRS M-EDL	11.4	7.6	11	5	17	13.8	8.9	12	8	18	
MDS-UPDRS ME	30.1	13.1	30	20	38	25.2	11.5	22	19	37	
MDS-UPDRS MC	3.0	2.6	4	1	4	2.7	3.0	2	0	4	
Lille Apathy Rating Scale	-23.7	7.1	-26	-28	-20	-24.8	5.9	-26	-28	-23	
Montgomery-Asberg Depression Rating Scale	9.5	5.3	9	6	13	11.5	5.4	12	8	15	
Parkinson Anxiety Scale	11.2	7.2	10	7	15	12.0	7.6	11	7	17	
LED	404.1	545.5	300	0	600	331.5	403.8	200	0	585	
Dopamine agonist LED	79.3	132.2	0	0	154	185.1	230.8	150	0	300	*p* < 0.05
Total LED	525.5	599.2	400	80	810	902.8	1826.0	550	154	900	

HYS = Hoehn-Yahr stages; LED = Levodopa equivalent dosage; MDS-UPDRS = Movement Disorders Society sponsored version of Unified Parkinson's Disease Rating Scale; MDS-UPDRS MC = motor complications (part IV of MDS-UPDRS); MDS-UPDRS ME = motor examination (part III of MDS-UPDRS); MDS-UPDRS M-EDL = motor experiences of daily living (part II of MDS-UPDRS); MDS-UPDRS nM-EDL = nonmotor experiences of daily living (part I of MDS-UPDRS); mMIDI = modified Minnesota Impulsive Disorders Interview; PDQ-39 SI = 39-item Parkinson's Disease Questionnaire Summary Index; UDysRS = Unified Dyskinesia Rating Scale (measured only on patients having dyskinesia, *n* = 50).

## Data Availability

The data used to support the findings of this study have not been made available because our ethical approval does not permit its deposition.

## Abbreviations

HYS: Hoehn-Yahr stages
ICDs: Impulse control disorders
LARS: Lille Apathy Rating Scale
LED: Levodopa equivalent dosage
MADRS: Montgomery-Asberg Depression Rating Scale
MDS-UPDRS: Movement Disorders Society Sponsored version of Unified Parkinson's Disease Rating Scale
MDS-UPDRS MC: Motor complications (part IV of MDS-UPDRS)
MDS-UPDRS ME: Motor examination (part III of MDS-UPDRS)
MDS-UPDRS M-EDL: Motor experiences of daily living (part II of MDS-UPDRS)
MDS-UPDRS nM-EDL: Nonmotor experiences of daily living (part I of MDS-UPDRS)
mMIDI: Modified Minnesota Impulsive Disorders Interview
MoCA: Montreal Cognitive Assessment
PAS: Parkinson Anxiety Scale
PD: Parkinson's disease
PD-ICD: Parkinson's disease patients with impulse control disorders
PIUQ: Problematic Internet Use Questionnaire
QUIP: Questionnaire for Impulsive-Compulsive Disorders in Parkinson's Disease
Y-BOCS-Internet: Yale-Brown Obsessive Compulsive Scale adapted for Internet use.

## Supplementary Materials

Supplementary Materials The supplementary file contains a table demonstrating each patient with impulse control disorders identified by the standard and standard+PIUQ methods.
