# Supplementary material for: Screening for Problematic Internet Use May Help Identify Impulse Control Disorders in Parkinson's Disease
Source: Behav Neurol. 2019 Feb 3;2019:4925015. doi: 10.1155/2019/4925015 (PMC6378069; doi:10.1155/2019/4925015)
Supplement: Supplementary Materials — The supplementary file contains a table demonstrating each patient with impulse control disorders identified by the standard and standard+PIUQ methods. [file 4925015.f1.pdf]

Supplementary file

Table. Impulse control disorders identified by the standard and standard+PIUQ methods.

	Compulsive gambling	Compulsive sexuality	Compulsive buying	Compulsive Eating	Compulsive hobbyism, punding, etc.	Drug addiction	Internet addiction	Internet overuse	Identified by the standard methods	Identified by the standard +internet method
Patient 1	S							I	S	
Patient 2	S		S					I	S	
Patient 3	S	S	S					I	S	
Patient 4	I							I		I
Patient 5	I							I		I
Patient 6	I							I		I
Patient 7	I				I		I	I		I
Patient 8	I	I						I		I
Patient 9	I	I	I		I		I	I		I
Patient 10		S	S		S			I	S	
Patient 11		I						I		I
Patient 12		I			I		I	I		I
Patient 13		I			I		I	I		I
Patient 14		I			I		I	I		I
Patient 15			S	S	S			I	S	
Patient 16				S				I	S	
Patient 17				S				I	S	
Patient 18				S					S	
Patient 19				S	S			I	S	
Patient 20				S				I	S	
Patient 21				S					S	
Patient 22				S				I	S	
Patient 23				S					S	
Patient 24			S					I	S	
Patient 25			S						S	
Patient 26			S		S			I	S	
Patient 27					S			I	S	
Patient 28					S			I	S	
Patient 29					S			I	S	
Frequency	9	2	7	9	12	0	5	25	19	10

Abbreviations: I = Internet use-based (standard+PIUQ) method; S = Standard method;

For definitions, refer to text.
